# High Ultra-Processed Food Consumption but No Association with Quality of Life in Saudi Children and Adolescents with ADHD

**DOI:** 10.3390/nu18182977

**Published:** 2026-09-11

**Authors:** Lina M. Alwaili, Norah S. Alsuhaibani, Heton K. Alotaibi, Loujain A. Alkhudairy, Hana A. Alanezi, Abeer A. Aljahdali, Dalal F. Alshamri, Abeer S. Alzaben

**Affiliations:** 1 Department of Health Sciences, College of Health and Rehabilitation Sciences, Princess Nourah bint Abdulrahman University, P.O. Box 84428, Riyadh 11671, Saudi Arabiansuhaibani1@gmail.com (N.S.A.); laalkhudairy@gmail.com (L.A.A.);; 2 Department of Clinical Nutrition, Faculty of Applied Medical Sciences, King Abdulaziz University, P.O. Box 80215, Jeddah 21589, Saudi Arabia; aaoaljahdali1@kau.edu.sa; 3 Department of Nutritional Sciences, University of Michigan, Ann Arbor, MI 48109, USA; 4 Department of Clinical Nutrition, King Abdullah bin Abdulaziz University Hospital, P.O. Box 84428, Riyadh 11564, Saudi Arabia

**Keywords:** attention-deficit hyperactivity disorder, quality of life, NOVA, ultra-processed food, adolescents

## Abstract

Background/Objectives: Attention-deficit hyperactivity disorder (ADHD) is a neurodevelopmental disorder characterized by inattention, hyperactivity, and impulsivity. Dietary interventions can potentially control ADHD symptoms, providing a non-pharmacological approach that may improve the quality of life (QoL). This cross-sectional study aimed to assess the association between ultra-processed food intake (UPF) and QoL in children and adolescents with ADHD in Saudi Arabia. Methods: A total of 128 participants (66% males and 33% females) diagnosed with ADHD, aged 8–18 years, were assessed. A validated Saudi food frequency questionnaire was used to assess dietary intake; the degree of food processing was assessed using a novel classification system. The health-related QoL was measured using the Pediatric Quality of Life Inventory. Results: Both the child (57.83 ± 18.12) and parent perspectives (57.97 ± 16.09) of QOL scores were lower than those of healthy norms across all domains. (*p*-value < 0.0001). The UPF represented the largest proportion of total energy intake (48.76 ± 13.40%), followed by unprocessed or minimally processed foods (39.80 ± 13.35%). Parent- and child-reported QoL scores were strongly correlated (*p* < 0.0001); both were significantly lower than the healthy normative values across all domains (*p* < 0.0001). Higher parental educational attainment was associated with better QoL scores from both the child’s and parents’ perspectives (*p* = 0.049 and *p* = 0.023, respectively). Conclusions: Children and adolescents with ADHD demonstrated high consumption of UPF and significantly lower QoL scores than healthy norms. No significant association was observed between UPF intake and QoL scores. Further studies are needed to better understand the factors influencing dietary quality and QoL among children and adolescents with ADHD.

## 1. Introduction

Attention-deficit hyperactivity disorder (ADHD) is a neurodevelopmental disorder commonly diagnosed in childhood that can persist into adulthood [1]. The global prevalence of ADHD ranges from 1.2% to 7.3% in adults, while in children it ranges from 5.9% to 7.1% [2]. The latest prevalence of ADHD in Makkah, Saudi Arabia, reached up to 9.2% [3]. Emerging evidence suggests that nutritional factors contribute to the development and severity of ADHD. Chronic deficiencies in essential minerals, including zinc, iron, magnesium, and iodine, and insufficient intake of long-chain polyunsaturated fatty acids have been associated with the development and worsening of ADHD symptoms in children [4]. Inadequate intake of omega-3 fatty acids, especially docosahexaenoic acid (DHA), may negatively affect brain development and function, potentially increasing susceptibility to ADHD and exacerbating symptom severity [4]. Furthermore, ADHD has been linked to unhealthy dietary patterns. Previous evidence showed that children with ADHD have lower adherence to the Mediterranean diet; higher consumption of sugar, candy, cola beverages, and fast food; and more frequent breakfast skipping compared with their healthy counterparts [5]. These findings suggest that unhealthy dietary habits may be associated with ADHD and could contribute to poor behavioral and health outcomes [5].

The management of ADHD primarily involves behavioral interventions, pharmacological treatments, or a combination of both [6]. However, concerns regarding the adverse effects of ADHD medication are common among both patients and parents. The common side effects of stimulant medications include headache, abdominal pain, insomnia, and decreased appetite, which may contribute to weight loss [6]. In addition, both stimulant and non-stimulant medications have been associated with adverse psychiatric effects, including mood disturbances and psychotic symptoms [7]. These treatment-related factors may negatively affect the quality of life (QoL) in children and adolescents with ADHD.

Previous studies have shown that children with ADHD experience significantly lower QoL than their healthy peers. A study conducted in the United Kingdom found that children with ADHD reported lower satisfaction with family relationships, sleep difficulties, challenges in daily activities and school performance, as well as greater behavioral and emotional problems [8]. ADHD has also been associated with anxiety, low self-esteem, impaired psychosocial functioning, and poor overall QoL [8]. Although previous research has shown that children with ADHD often consume more sugar, candy, soft drinks, and fast food and show lower adherence to healthy dietary patterns, the specific contribution of ultra-processed foods (as defined by the NOVA classification) to health-related quality of life has not been adequately investigated [4,5]. Moreover, no study has examined this association in Saudi children and adolescents with ADHD. Therefore, the present study aimed to assess the association between ultra-processed food (UPF) intake and QoL among children and adolescents with ADHD in Saudi Arabia.

## 2. Materials and Methods

### 2.1. Participants

A cross-sectional study was conducted among children and adolescents diagnosed with ADHD aged 8–18 years and living in Saudi Arabia. The exclusion criteria were disabilities, diseases that may affect dietary intake, absorption, and metabolism (e.g., celiac disease, allergies), or chronic diseases (e.g., diabetes mellitus, thyroid disorders, or cardiovascular disease). Participants were recruited from the Ishraq Association (Saudi ADHD Society), Riyadh, Saudi Arabia, and King Abdullah bin Abdulaziz University Hospital, Riyadh, Saudi Arabia, between January 2024 and December 2025.

This study was approved by the Institutional Review Boards of Princess Nourah bint Abdulrahman University, Riyadh, Saudi Arabia (IRB No. 23-0746) and King Abdullah bin Abdulaziz University Hospital (IRB No. RO-2025-P-015). Written informed consent was obtained from the parents or legal guardians of all participants, and written assent was obtained from the participating children and adolescents before enrolling in the study.

### 2.2. Research Instruments

After consent and assent were obtained, an online survey was shared with parents for completion. The participants were asked questions about their sociodemographic characteristics, anthropometric measurements, and medical history (e.g., duration since diagnosis and medication use). Weight (kg) and height (cm) were self-reported and used to calculate the participants’ body mass index (BMI) and *z*-scores using the Centers for Disease Control and Prevention (CDC) growth chart calculator for children aged 2–20 years on the Peditools website [9].

A validated Saudi Food Frequency Questionnaire (FFQ) was used to measure the dietary intake over the previous year [10]. The FFQ was then analyzed using the ESHA Food Processor software (version 11.7.1, Food and Nutrition Information Center, University of Washington, 2023) to estimate the daily calorie intake of each participant [11]. Consumption of UPFs was assessed using the FFQ and classified according to the NOVA Food Classification system [12,13,14,15]. The NOVA system categorizes foods into four groups based on the nature, purpose, and extent of industrial processing. The first group includes unprocessed or minimally processed foods, such as the natural edible parts of plants and animals that undergo minimal alterations. The second group comprises processed culinary ingredients extracted from food or nature, including oils, sugars, and salts, which are typically used in food preparation. The third group comprises processed foods produced by adding culinary ingredients to minimally processed foods such as canned products, cheeses, and freshly made bread. The fourth group includes ultra-processed foods, which are industrial formulations manufactured using multiple ingredients and additives and are often designed for convenience, palatability, and longer shelf life [12,13,14,15].

The Pediatric Quality of Life Inventory measurement model (PedsQL) was used to measure health-related QoL in this study [16,17,18]. This model has 4 multidimensional scales (physical, emotional, social, and school functioning). The physical health scale contained 8 questions, while each of the emotional, social, and school functioning scales contained 5 questions, with a total of 23 questions for the survey. The PedsQL has different versions according to age group. In this study, the ages 8–12 and 13–18 versions were used. Moreover, this model has two different versions for QoL to be reported from the child’s and parent’s perspectives. The answers to the questions are on a scale from 0 to 4, where 0 means “It is never a problem” and 4 means “It is always a problem.” When scoring this model, items are reverse-scored, and a 0–4 scale is transformed to a 0–100 scale for ease of interpretability. The transformation was as follows: 0 = 100, 1 = 75, 2 = 50, 3 = 25, and 4 = 0. Higher scores indicate improved QoL, with a maximum score of 100 and a minimum score of 0. The total score was calculated as the mean of all participants’ responses to questionnaire items.

### 2.3. Statistical Analysis

No formal a priori sample-size calculation was conducted, as the study was exploratory and aimed to assess associations rather than test a pre-specified effect size. The final analytic sample of 128 participants was determined by the number of eligible children and adolescents recruited during the study period after applying the exclusion criteria for implausible energy intake and anthropometric data. Data were analyzed using Statistical Analysis System (SAS) 9.4 TS1M7 (SAS Institute Inc., Cary, NC, USA). Descriptive analyses were conducted and presented as frequencies and percentages or as mean ± standard deviation for categorical and continuous variables, respectively. The distribution of continuous variables was visually assessed by evaluating histograms and QQ plots. A one-sample *t*-test was used to compare the quality-of-life scores with values reported for healthy norms [16]. Pearson’s correlation was used to examine the strength and direction of the linear relationship between two continuous variables that were normally distributed. Spearman’s rank correlation was used to evaluate the strength and direction of monotonic relationships between two continuous variables that were not normally distributed. Due to the missing values in the anthropometric measurements, pairwise deletion was applied in the bivariate correlation analyses. All analyses were limited to bivariate associations with no adjustment to any confounding factor. All tests were two-tailed, and statistical significance was considered as a *p*-value < 0.05.

## 3. Results

**Figure 1:**
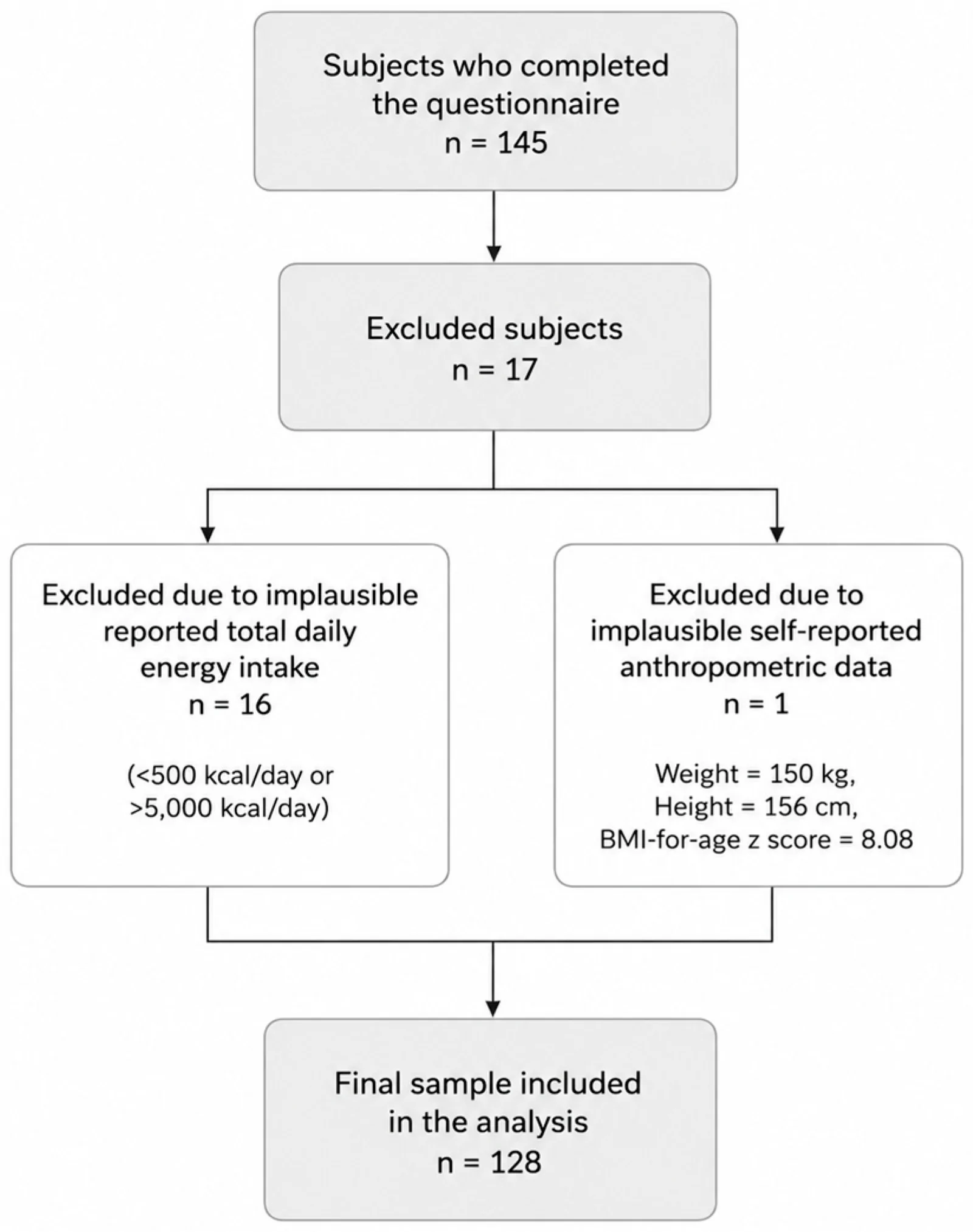
Flow chart of participant inclusion and exclusion.

A total of 145 subjects completed the study; however, 16 subjects were excluded because of implausibly reported total daily energy intake, which was either less than 500 or >5000 calories [19]. In addition, one subject was excluded because of implausible self-reported anthropometric data (weight = 150 kg, height = 156 cm, resulting in a BMI-for-age *z*-score of 8.08) (Figure 1).

### 3.1. Sociodemographic

Table 1 presents the sociodemographic data of the participants. The study sample included 128 children and adolescents diagnosed with ADHD living in Saudi Arabia, with an average age of 11.51 ± 3.07 years. Male participants represented 85 (66.41%) of the sample, and 73.44% of the sample was reported to reside in the central region. Mothers and fathers had a range of educational backgrounds, with the largest proportion holding a bachelor’s degree (mothers: 64.06%; fathers: 55.47%). The most commonly reported household monthly income group falls between 7000 and 15,000 SAR (33.59%). Furthermore, 47.66% of the participants reported using medications for ADHD. The mean anthropometric measures are 0.02 ± 1.43 for weight-for-age *z*-score, −0.41 ± 1.34 for height-for-age *z*-score, and 0.22 ± 1.59 for BMI-for-age *z*-score.

### 3.2. Health-Related QoL Data

Table 2 summarizes the average scores for both child and parent perspectives on the child’s QoL for the four key domains of well-being: physical, social, emotional, and school. Children’s reported values were very similar to the numbers reported by their parents; the correlation was positive and strong (r > 0.80, *p*-value < 0.0001). The average and standard deviation for the total score reported by parents are 57.97 ± 16.09, ranging from 17.39 to 91.30, while the total score reported by children and adolescents is 57.83 ± 18.12, ranging from 10.87 to 100.00. All domains ranged from 0 to 100, except for the physical functioning score, which ranged from 12.50 to 100. The scores reported by parents, children, and adolescents were significantly lower than the scores for healthy norms (*p*-value < 0.0001).

Table 3 illustrates the distribution of the mean health-related QoL scores among children and adolescents with ADHD and their parents across different descriptive variables. Regarding parental education, statistically significant differences were found in higher education levels, correlating with higher mean health-related QoL scores in both groups. The mother’s level of education was associated with higher scores for children and adolescents (*p*-value = 0.0274) and parents (*p*-value = 0.0235). Similarly, fathers’ education levels were associated with higher scores for children and adolescents (*p*-value = 0.0490) and parents (*p*-value = 0.0234). No statistically significant differences were noted in the total score according to sex, respondent type, residence, living situation, income, number of siblings, ADHD medication use, medication type, or age at ADHD diagnosis among the children, adolescents, or their parents.

Table 4 shows the bivariate association between health-related QoL scores, age, and anthropometric measures. The data showed that the BMI *z*-score was inversely associated with school functioning scores reported by both children and adolescents (r = −0.244; *p*-value = 0.0084) and their parents (r = −0.236; *p*-value = 0.0108). In addition, the BMI *z*-score was inversely associated with the total health-related QoL scores reported by parents (r = −0.224; *p*-value = 0.0155). No significant correlations were observed among age, weight, or height *z*-scores.

### 3.3. Ultra-Processed Food Intake

**Figure 2:**
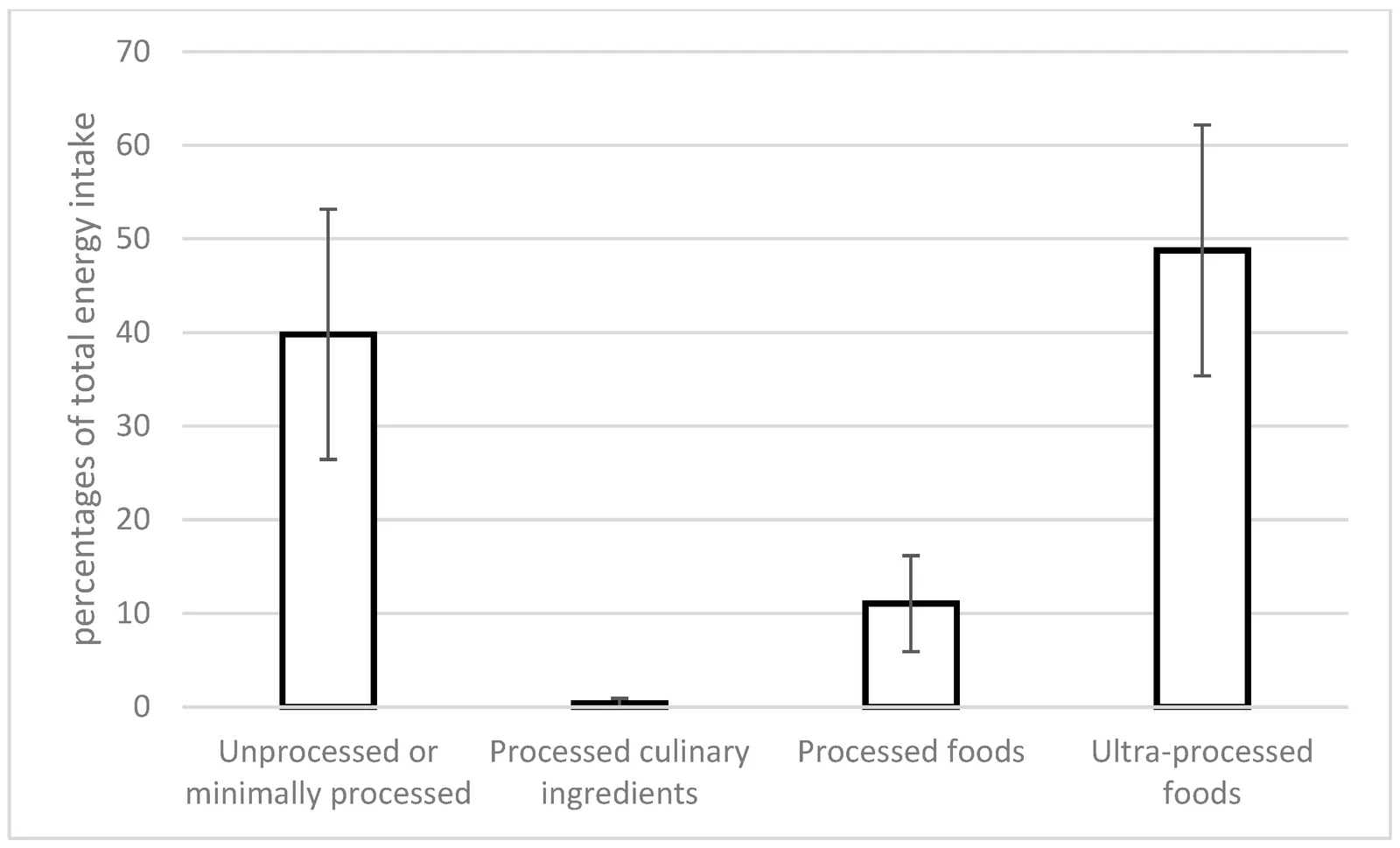
Description of the intake of NOVA scores among children and adolescents with ADHD. This chart depicts the average daily food consumption patterns of children with ADHD in the study sample (N = 128), categorized by the NOVA classification system.

Figure 2 illustrates the distribution of the means and standard deviations of the NOVA scores, expressed as percentages of the total energy intake. Ultra-processed foods (UPFs) accounted for 48.76% ± 13.40, which represents the highest contribution, followed by unprocessed or minimally processed foods (UFs) at 39.80% ± 13.35. The mean share for processed foods (PFs) was 11.05 ± 5.13, and for processed culinary ingredients (PCIs) was 0.38 ± 0.56. Nutritional profiles of the study population are presented in Supplementary Table S1.

Table 5 presents the mean ± standard deviation of the percentage of TEI from UPFs for various variables. The results revealed no statistically significant differences in mean UPF intake across all demographic or ADHD-related variables (all *p*-values > 0.05).

Table 6 shows the bivariate association between the percentage of TEI from UPFs, age, and several anthropometric measures. The data showed that the BMI *z*-score was inversely associated with the percentage of TEI in UPFs (r = −0.183; *p*-value =0.0494). No significant correlations were observed among age, weight, or height *z*-scores.

## 4. Discussion

To our knowledge, this is the first cross-sectional study to examine the association between UPF intake and QoL in children and adolescents with ADHD in Saudi Arabia. The intake of UPFs among the study population was high, accounting for 48.76% of the average daily caloric intake. Moreover, the QoL scores in this population were significantly lower than those of healthy norms across all domains from both children’s and parents’ perspectives. Maternal and paternal education were associated with QoL. However, there were no associations between UPF intake, sociodemographic and anthropometric variables, or QoL.

This study examined the association between UPF intake and the QoL of children and adolescents with ADHD in Saudi Arabia. The intake of UPF among the study population was high, accounting for 48.76% of the average daily caloric intake. Moreover, the QoL scores in this population were significantly lower than those of healthy norms across all domains from both children’s and parents’ perspectives. Mothers’ and fathers’ education was associated with QOL. However, no associations were noted among UPF intake, sociodemographic and anthropometric variables, or QoL.

Moreover, the majority of participants consumed diets primarily composed of UPFs (49%). However, a significant proportion (40%) originated from unprocessed or minimally processed foods. These findings are consistent with those reported by Al-Muammar et al. (2015), who found that Saudi children diagnosed with ADHD frequently consumed foods high in additives and preservatives, including coloring agents and simple sugars, with 51.30% reporting processed food consumption as snacks three to four times per week [20]. Similarly, another study found that children with ADHD had a lower consumption of fruits, vegetables, pasta, and rice, along with a higher intake of sugar, candy, cola beverages, and non-cola soft drinks, compared with healthy controls [5]. Comparable findings were also reported by Akin et al. (2022), who demonstrated that children with ADHD consumed greater amounts of processed meat products, dairy-based desserts, confectionery items, and foods rich in saturated fat and sugar than the healthy controls [21]. When comparing our findings with the previous literature, important methodological differences must be considered. Earlier studies often assessed specific food items (e.g., sugar, candy, soft drinks, or processed meats) rather than applying systematic processing-level classification such as NOVA. In addition, some studies used different dietary assessment methods (e.g., 24 h recalls or food diaries) that may capture short-term intake more accurately than an FFQ designed to estimate habitual intake over the previous year.

The current study revealed no statistically significant differences in the mean UPF intake across any sociodemographic variable. These findings contrast with some existing literature. For example, Huberts-Bosch et al. (2024) suggested that children with ADHD whose parents had higher education levels tended to consume healthier diets [22]. Similarly, Fuentes-Albero (2019) found a positive association among parental education, children’s nutritional knowledge, and dietary habits [23]. Although it is often hypothesized that higher education leads to better nutritional role modeling and knowledge, the data in this study suggest a more homogeneous pattern of consumption.

The current study found that children and adolescents with ADHD perceived QoL score was lower than the healthy norms, and the highest QoL scores were observed in the physical domain for both child and parent reports. This finding is consistent with a study conducted in Saudi Arabia that found higher QoL scores in the physical functioning domain as reported by both children and their parents [24]. The lowest QoL scores were found in the school domain in both the children’s and parents’ reports [24,25,26]. Consistent with these findings, the current study also identified school domains as the least rated aspects of QoL across both child and parent reports, further reinforcing the indication that academic environments may present the most significant challenges to the well-being of this population.

The findings of the current study differ from those reported by Rocco et al. (2021), who identified an association between longer ADHD duration, greater symptom severity, treatment initiation, and lower QoL scores [27]. The lack of improvement in QoL following treatment may be explained by the inclusion of children with more severe baseline symptoms, which may have contributed to poorer QoL outcomes. In contrast, the current study found no significant differences between time since diagnosis, medication use, and QoL. This discrepancy may be attributed to differences in sample characteristics, treatment approaches, or timing of QoL assessment relative to treatment initiation. Similarly, no association was found between ADHD medication use and QoL in the present study. However, other studies have reported positive associations between medication use and better QoL among children and adolescents with ADHD, particularly regarding emotional/behavioral functioning, mental health, and self-esteem [28,29]. These findings highlight the complexity of the relationship between medication use and QoL and suggest that treatment effects vary depending on symptom severity, treatment type, and duration.

This study did not find an association between sociodemographic data and QoL in children or adolescents diagnosed with ADHD. This finding contrasts with that of Riley et al. (2006), who reported poorer QoL among children with ADHD who were not living with both parents [30]. This discrepancy may be due to cultural variations in family structures. In Saudi Arabia, extended family networks often play a significant supportive role and may help buffer the impact of not living with both parents, unlike the predominantly nuclear-family patterns observed in many Western studies [31,32].

Investigating the relationship between UPF intake and QoL in children and adolescents with ADHD remains challenging. Although the current study found no significant association between UPF intake and QoL, the BMI-for-age *z*-score was inversely associated with school functioning in both the child and parent reports. In contrast, Park et al. (2012) provide a definitive stance on the link between diet and ADHD along with learning disabilities in school-aged children [33]. The study suggested that a high intake of salt, sweetened desserts, and fried foods was associated with the exacerbation of learning, attention, and behavioral difficulties. This study suggested that specific dietary behaviors have a direct correlation with the severity and presence of ADHD symptoms and learning disabilities [33]. Similarly, a study of 3206 Chinese adolescents found that the consumption of specific ultra-processed food items, including instant noodles, sugary beverages, and fried foods, was associated with poorer QoL, whereas fruit intake was associated with better QoL outcomes [34]. The current study measured QoL using the validated PedsQL in the present study, whereas other investigations employed different instruments or focused primarily on ADHD symptom severity rather than multidimensional QoL. The differences in exposure and outcome assessment may explain the lack of association observed between UPF intake and QoL in our cohort.

Certain limitations of the current study should be acknowledged. The relatively small sample size may have prevented detecting subtle associations between dietary factors and the study’s outcomes, increasing the possibility of Type II error. Furthermore, due to the small sample size, the study’s conclusions are not generalizable to all children and adolescents diagnosed with ADHD; thus, future studies with larger sample size are warranted studies with greater statistical power are warranted to characterize the dietary habits among children and adolescents diagnosed with ADHD. The lack of adjustment for potential confounding factors is another limitation in the current study. ADHD symptom severity was not assessed. Clinical severity is a major potential confounding variable, as children and adolescents with more severe hyperactivity or impulsivity may be more likely to consume higher amounts of ultra-processed foods while simultaneously experiencing lower quality of life due to greater functional impairment. The lack of stratification by symptom severity may therefore have attenuated or masked true associations between dietary intake and quality of life. Future studies should incorporate validated, objective rating scales to allow for severity-adjusted analyses. In addition, anthropometric and dietary data were self-reported by parents, which introduces the potential for measurement error and reporting bias. Missing values for height and weight were handled using pairwise deletion in the bivariate correlation analyses. Nevertheless, this study has several notable strengths. Health-related QoL was comprehensively assessed using the validated PedsQL from both the child and parent perspectives, allowing for a broader evaluation of QoL outcomes. In addition, dietary intake was assessed using the FFQ, whereas the NOVA classification system was used to evaluate diet quality and UPF consumption.

## 5. Conclusions

Understanding the factors that influence the QoL of children and adolescents with ADHD is important. This study provides valuable insights by demonstrating that children and adolescents with ADHD experience significantly lower QoL across all domains than the healthy norms. The consumption of UPF was high in the study population (49%), and the second highest was unprocessed food, which constituted 40% of total calorie intake. Previous studies have found high consumption of unhealthy and processed foods among children and adolescents with ADHD, which is associated with poor ADHD outcomes and QoL [33,34]. However, the current study did not observe an association between dietary choices and QoL, which could be due to the use of the FFQ, which might lead to over-or underreporting of food intake. Another possible reason is the sample size and characteristics. ADHD symptom severity was not assessed in this study, and severe symptoms may have masked the effect of dietary intake. These findings suggest that while diet may not be the sole contributor, it warrants further investigation of its role alongside other potential factors influencing QoL in this population.

## Figures and Tables

**Table 1:** Sociodemographic characteristics of children and adolescents with ADHD (N = 128).

Variable/Category	N (%) or Mean ± SD
**Age (years)**	11.51 ± 3.07
**Height (cm) ^1^**	142.92 ± 21.22
**Weight (kg) ^2^**	42.80 ± 18.36
**Weight-for-age** ***z*** **-score ^2^**	0.02 ± 1.43
**Height-for-age** ***z*** **-score ^3^**	−0.41 ± 1.34
**BMI-for-age** ***z*** **-score ^3^**	0.22 ± 1.59
**Sex**	
Male	85 (66.41%)
Female	43 (33.59%)
**Respondent type**	
The participant	14 (10.94%)
Mother	93 (72.66%)
Father	16 (12.50%)
Others	5 (3.91%)
**Residence**	
Central	94 (73.44%)
Eastern	11 (8.59%)
Northern	1 (0.78%)
Western	19 (14.84%)
Southern	3 (2.34%)
**Living with**	
Parents	99 (77.34%)
Mother	29 (22.66%)
**Mother education**	
Below high school	8 (6.25%)
High school	15 (11.72%)
Bachelor’s degree	82 (64.06%)
Higher education	23 (17.97%)
**Father education**	
Below high school	7 (5.47%)
High school	30 (23.44%)
Bachelor’s degree	71 (55.47%)
Higher education	20 (15.63%)
**Household income (SR)**	
Less than 7000	24 (18.75%)
7000–15,000	43 (33.59%)
16,000–20,000	32 (25.00%)
More than 20,000	29 (22.66%)
**Number of siblings**	
None	7 (5.47%)
1–2	56 (43.75%)
3–4	51 (39.84%)
More than 4	14 (10.94%)
**ADHD medication use**	
No	67 (52.34%)
Yes	61 (47.66%)
**ADHD medication Type**	
Atomoxetine	15 (11.72%)
Methylphenidate	37 (28.91%)
Others	15 (11.72%)
**Age at ADHD Diagnosis ^4^**	
Less than 1 year	20 (15.63%)
1 year ago	15 (11.72%)
2 years ago	19 (14.84%)
More than 2 years	68 (53.13%)

Mean and standard deviation or frequency and percentages were presented for continuous and categorical variables, respectively. ^1^ N = 117; missing 11 observations. ^2^ N = 121; missing 7 observations. ^3^ N = 116; missing 12 observations. ^4^ N = 122; missing 6 observations.

**Table 2:** Health-related quality of life data in children and adolescents with ADHD, their parents, and comparison with healthy norms (N = 128).

Domain	Parents			Children and Adolescents		
	Mean ± SD	Healthy Norms ^1^	*p*-Value	Mean ± SD	Healthy Norms ^1^	*p*-Value
Emotional functioning score	51.02 ± 25.48	81	**<0.0001**	49.77 ± 22.20	79	**<0.0001**
Physical functioning score	71.92 ± 22.60	84	**<0.0001**	72.78 ± 20.03	88	**<0.0001**
School functioning score	48.09 ± 24.78	78	**<0.0001**	48.83 ± 22.42	81	**<0.0001**
Social functioning score	51.84 ± 29.26	83	**<0.0001**	51.64 ± 26.68	85	**<0.0001**
Total score ^2^	57.97 ± 16.09	82	**<0.0001**	57.83 ± 18.12	84	**<0.0001**

Values are presented as Mean ± SD. ^1^ The mean of healthy norms was obtained from Varni et al. (2003) [16]. ^2^ Total values were computed as the mean of four domains: physical, emotional, social, and school functioning. *p*-values were calculated based on a one-sample *t*-test. *p*-values less than 0.05 are bolded.

**Table 3:** Health-related quality of life data in children and adolescents with ADHD and their parents across different descriptive variables (N = 128).

Variable	N	Children and Adolescents ^1^		Parents ^1^	
		Mean ± SD	*p*-Value	Mean ± SD	*p*-Value
**Sex**					
Male	85	58.44 ± 15.93	0.6467	58.66 ± 17.56	0.4696
Female	43	57.05 ± 16.55		56.19 ± 19.29	
**Respondent type**					
The participant	14	56.75 ± 17.97	0.2435	59.86 ± 22.21	0.0844
Mother	93	56.76 ± 15.90		55.63 ± 17.63	
Father	16	65.49 ± 12.73		67.87 ± 14.29	
Others	5	60.00 ± 21.77		60.87 ± 19.03	
**Residence**					
Central	94	58.35 ± 15.61	0.7427	58.24 ± 18.12	0.8536
Eastern	11	55.73 ± 22.31		53.46 ± 22.26	
Northern	1	76.09 ± 0.00		71.74 ± 0.00	
Western	19	57.44 ± 14.90		58.07 ± 15.56	
Southern	3	51.81 ± 18.90		54.71 ± 26.30	
**Living with**					
Parents	99	58.08 ± 16.20	0.8901	57.86 ± 17.96	0.9710
Mother	29	57.61 ± 15.96		57.72 ± 18.99	
**Mother education**					
Below high school	8	42.12 ± 13.94	**0.0274**	40.08 ± 12.52	**0.0235**
High school	15	60.87 ± 11.50		61.88 ± 11.92	
Bachelor’s degree	82	59.35 ± 15.58		59.38 ± 18.32	
Higher education	23	56.71 ± 18.66		55.81 ± 19.49	
**Father education**					
Below high school	7	50.16 ± 20.61	**0.0490**	48.60 ± 23.53	**0.0234**
High school	30	53.15 ± 15.60		52.03 ± 16.43	
Bachelor’s degree	71	58.99 ± 16.11		58.85 ± 17.71	
Higher education	20	64.35 ± 12.73		66.14 ± 17.07	
**Household income**					
Less than 7000	24	54.44 ± 17.13	0.0818	53.94 ± 18.39	0.1690
7000–15,000	43	57.05 ± 13.89		56.62 ± 16.49	
16,000–20,000	32	55.91 ± 15.51		56.59 ± 19.12	
More than 20,000	29	64.54 ± 17.76		64.21 ± 18.44	
**Number of siblings**					
None	7	60.56 ± 17.15	0.7542	59.32 ± 18.25	0.8666
1–2	56	59.14 ± 15.67		58.62 ± 18.32	
3–4	51	56.10 ± 16.54		56.20 ± 18.14	
More than 4	14	58.85 ± 16.63		59.86 ± 18.71	
**ADHD medication use**					
No	67	56.33 ± 15.95	0.2262	55.97 ± 16.85	0.2251
Yes	61	59.78 ± 16.17		59.87 ± 19.35	
**ADHD medication type**					
Atomoxetine	15	54.93 ± 13.71	0.7796	54.49 ± 15.85	0.9246
Methylphenidate	37	57.58 ± 17.64		56.23 ± 18.23	
Others	15	54.64 ± 14.25		56.81 ± 15.14	
**Age at ADHD diagnosis ^2^**					
Less than 1 year	20	59.57 ± 16.19	0.7722	58.42 ± 16.63	0.6020
1 year ago	15	57.17 ± 14.61		57.03 ± 19.45	
2 years ago	19	60.58 ± 19.84		60.58 ± 23.04	
More than 2 years	68	56.68 ± 15.73		56.14 ± 17.32	

Values are presented as Mean ± SD. ^1^ Total values were computed as the mean of four domains: physical, emotional, social, and school functioning. ^2^ N = 122; 6 observations were missing. *p*-values were calculated based on an independent-samples *t*-test for binary variables and one-way ANOVA for categorical variables with more than two levels of response. *p*-values less than 0.05 are bold.

**Table 4:** Correlation coefficients between health-related quality of life scores in children and adolescents with ADHD and their parents across different descriptive variables (N = 128).

Variable	Age	*p*-Value	Weight-for-Age *z*-Score ^1^	*p*-Value	Height-for-Age *z*-Score ^2^	*p*-Value	BMI-for-Age *z*-Score ^2^	*p*-Value
Child/adolescent-reported score for health-related quality of life ^3^	−0.036	0.6891	−0.091	0.3192	0.118	0.2090	−0.174	0.0617
Emotional functioning score	−0.111	0.2130	−0.014	0.8785	0.128	0.1708	−0.103	0.2714
Physical functioning score ^4^	0.031	0.7297	−0.068	0.4575	0.048	0.6077	−0.106	0.2575
School functioning score	−0.111	0.2133	−0.159	0.0825	0.108	0.2476	−0.244	**0.0084**
Social functioning score	0.095	0.2886	0.055	0.5466	0.121	0.1944	0.008	0.9293
Parent-reported score for health-related quality of life ^3^	−0.057	0.5226	−0.106	0.2476	0.139	0.1378	−0.224	**0.0155**
Emotional functioning score	−0.075	0.3975	−0.026	0.7769	0.173	0.0639	−0.151	0.1045
Physical functioning score ^4^	0.035	0.6950	−0.053	0.5624	0.047	0.6180	−0.123	0.1869
School functioning score	−0.144	0.1038	−0.145	0.1124	0.097	0.2997	−0.236	**0.0108**
Social functioning score	0.074	0.4066	0.022	0.8122	0.160	0.0869	−0.065	0.4852

^1^ N = 121; missing 7 observations. ^2^ N = 116; missing 12 observations. ^3^ Total values were computed as the mean of four domains: physical, emotional, social, and school functioning. ^4^ The Spearman rank correlation coefficient is presented. Otherwise, the Pearson correlation coefficient is presented. *p*-values less than 0.05 are bold.

**Table 5:** Description of the intake of ultra-processed foods across different descriptive variables (N = 128).

Variable/Category	N	Mean ± SD ^1^	*p*-Value
**Sex**			
Male	85	48.94 ± 12.89	0.8344
Female	43	48.41 ± 14.50	
**Respondent type**			
The participant	14	48.12 ± 14.99	0.9714
Mother	93	48.61 ± 13.74	
Father	16	50.25 ± 9.65	
Others	5	48.72 ± 16.32	
**Residence**			
Central	94	49.24 ± 13.22	0.8278
Eastern	11	49.58 ± 11.36	
Northern	1	36.34 ± 0.00	
Western	19	47.17 ± 13.06	
Southern	3	45.05 ± 30.94	
**Living with**			
Parents	99	48.28 ± 14.03	0.4525
Mother	29	50.41 ± 11.00	
**Mother education**			
Below high school	8	44.67 ± 19.14	0.9239
High school	15	48.47 ± 13.85	
Bachelor’s degree	82	48.65 ± 13.35	
Higher education	23	50.15 ± 11.48	
**Father education**			
Below high school	7	44.63 ± 20.40	0.4266
High school	30	51.88 ± 13.29	
Bachelor’s degree	71	48.43 ± 13.08	
Higher education	20	46.71 ± 11.91	
**Household income**			
Less than 7000	24	46.28 ± 11.64	0.3295
7000–15,000	43	49.26 ± 14.86	
16,000–20,000	32	46.84 ± 13.16	
More than 20,000	29	52.21 ± 12.56	
**Number of siblings**			
None	7	46.17 ± 16.59	0.7294
1–2	56	50.16 ± 11.94	
3–4	51	47.51 ± 14.73	
More than 4	14	49.04 ± 13.04	
**ADHD medication use**			
No	61	48.77 ± 12.49	0.9021
Yes	67	48.76 ± 14.26	
**ADHD medication type**			
Atomoxetine	15	50.79 ± 16.31	0.7309
Methylphenidate	37	48.80 ± 14.40	
Others	15	46.61 ± 12.25	
**Age at ADHD diagnosis ^2^**			
Less than 1 year	20	49.83 ± 12.37	0.7917
1 year ago	15	51.42 ± 18.57	
2 years ago	19	48.37 ± 12.57	
More than 2 years	68	47.82 ± 12.85	

^1^ Expressed as percentages of total energy intake. ^2^ N = 122; 6 observations missing. *p*-values were calculated based on an independent-samples *t*-test for binary variables and one-way ANOVA for categorical variables with more than two levels of response.

**Table 6:** Correlation coefficients between ultra-processed food intake and quality of life among children and adolescents with ADHD (N = 128).

Variable	Correlation Coefficients (r)	*p*-Value
**Child/adolescent-reported score for health-related quality of life ^1^**	0.016	0.8542
Emotional functioning score	0.133	0.1346
Physical functioning score ^2^	−0.026	0.7675
School functioning score	−0.019	0.8353
Social functioning score	−0.069	0.4419
**Parent-reported score for health-related quality of life**	0.034	0.7060
Emotional functioning score	0.060	0.5034
Physical functioning score ^2^	−0.027	0.7637
School functioning score	−0.045	0.6173
Social functioning score	0.057	0.5222
**Age**	−0.016	0.8539
**Weight-for-age** ***z*** **-score ^3^**	−0.089	0.3299
**Height-for-age** ***z*** **-score ^4^**	0.019	0.8417
**BMI-for-age** ***z*** **-score ^4^**	−0.183	0.0494

^1^ Total values were computed as the mean of four domains: physical, emotional, social, and school functioning. ^2^ Spearman rank correlation coefficient is presented. Otherwise, the Pearson correlation coefficient is presented. ^3^ N = 121; missing 7 observations. ^4^ N = 116; missing 12 observations.

## Data Availability

The data presented in this study are not publicly available due to ethical and privacy restrictions. In accordance with the informed consent obtained from participants, the data are accessible only to the research team.

## Supplementary Materials

The following supporting information can be downloaded at: https://www.mdpi.com/article/10.3390/nu18182977/s1, Table S1: Nutritional profile among children and adolescents with ADHD (N = 128).

## Abbreviations

The following abbreviations are used in this manuscript:

ADHD	Attention-Deficit Hyperactivity Disorder
UPF	Ultra-Processed Food
QoL	Quality of Life
FFQ	Food Frequency Questionnaire
TEI	Total Energy Intake
