# Supplementary material for: High Ultra-Processed Food Consumption but No Association with Quality of Life in Saudi Children and Adolescents with ADHD
Source: Nutrients. 2026 Sep 11;18(18):2977. doi: 10.3390/nu18182977 (PMC13610857; doi:10.3390/nu18182977)
Supplement: Supplementary file 1 [file nutrients-18-02977-s001.zip › nutrients-4538984-supplementary.pdf]

Supplementary Table S1: Nutritional profile among children and adolescents with ADHD (N=128):

	Mean ± SD
**Calories (kcal)**	2316.70 ± 1035.30
**Carbohydrate (kcal)**	1135.01 ± 530.97
**Percent carbohydrate**	48.94 ± 6.17
**Protein (kcal)**	419.22 ± 209.85
**Percent protein**	18.15 ± 4.02
**Fat (kcal)**	768.72 ± 359.42
**Percent fat**	33.21 ± 4.10
**Saturated fatty acids (kcal)**	275.84 ± 139.13
**Percent SFA**	11.84 ± 2.33
**Trans fatty acids (kcal)**	2.37 ± 2.68
**Percent TFA**	0.10 ± 0.10
**Monounsaturated fatty acids (kcal)**	234.19 ± 112.94
**Percent MUFA**	10.23 ± 2.07
**Polyunsaturated fatty acids (kcal)**	125.25 ± 61.08
**Percent PUFA**	5.46 ± 1.13
**Fiber (g/1000 kcal)**	10.83 ± 2.95
**Added sugars (mg/1000 kcal)**	15.61 ± 8.82
**Total sugars (mg/1000 kcal)**	48.25 ± 13.49
**Cholesterol (mg/1000 kcal)**	194.34 ± 91.89
**Sodium (mg/1000 kcal)**	1334.18 ± 208.31
**Iron (mg/1000 kcal)**	7.88 ± 2.04
**Calcium (mg/1000 kcal)**	596.27 ± 222.75
**Magnesium (mg/1000 kcal)**	115.82 ± 21.65
**Phosphorus (mg/1000 kcal)**	582.80 ± 127.60
**Potassium (mg/1000 kcal)**	1207.58 ± 259.37
**Vitamin D (IU/1000 kcal)**	91.82 ± 59.06

**Vitamin D (µg/1000 kcal)**	2.30 ± 1.48
**Zinc (mg/1000 kcal)**	4.59 ± 1.20
**Copper (µg/1000 kcal)**	0.71 ± 0.54
**Manganese (mg/1000 kcal)**	0.71 ± 0.40
**Selenium (µg/1000 kcal)**	50.44 ± 15.61
**Vitamin A (IU/1000 kcal)**	2539.15 ± 2356.52
**Vitamin A (RAE/1000 kcal)**	473.65 ± 363.95
**Carotene (µg/1000 kcal)**	176.37 ± 211.46
**Retinol (µg/1000 kcal)**	340.09 ± 354.48
**Beta-carotene (µg/1000 kcal)**	1364.97 ± 1289.75
**Vitamin B1 (mg/1000 kcal)**	0.70 ± 0.14
**Vitamin B2 (mg/1000 kcal)**	0.97 ± 0.26
**Vitamin B3 (mg/1000 kcal)**	10.95 ± 3.07
**Vitamin B3 NE (mg/1000 kcal)**	12.96 ± 3.68
**Vitamin B6 (mg/1000 kcal)**	0.83 ± 0.18
**Vitamin B12 (µg/1000 kcal)**	3.70 ± 2.68
**Folate (µg/1000 kcal)**	206.24 ± 43.99
**Folate DFE (µg/1000 kcal)**	227.41 ± 49.14
**Biotin (µg/1000 kcal)**	5.05 ± 3.65
**Vitamin C (mg/1000 kcal)**	44.90 ± 27.42
**Vitamin E (mg/1000 kcal)**	4.08 ± 1.13
**Vitamin K1 (µg/1000 kcal)**	40.97 ± 23.28
**Caffeine (mg/1000 kcal)**	19.85 ± 18.19
**Pantothenic acid (mg/1000 kcal)**	1.15 ± 0.45
**Chromium (µg/1000 kcal)**	0.94 ± 0.71
**Fluoride (mg/1000 kcal)**	0.11 ± 0.12
**Iodine (µg/1000 kcal)**	13.57 ± 11.92
**Molybdenum (µg/1000 kcal)**	6.01 ± 6.05
**Omega-3 (g/1000 kcal)**	0.57 ± 0.12

**Omega-6 (g/1000 kcal)**	5.24 ± 1.19
**Choline (mg/1000 kcal)**	183.44 ± 74.97

Values are presented as Mean ± SD.
kcal = kilocalories; SFA = saturated fatty acids; MUFA = monounsaturated fatty acids; PUFA = polyunsaturated fatty acid
